# Soft and Hard Tissue Regeneration—A Special Issue of Dentistry Journal

**DOI:** 10.3390/dj6010004

**Published:** 2018-01-23

**Authors:** Carlos E. Nemcovsky, Miron Weinreb

**Affiliations:** 1 Department of Periodontology and Dental Implantology, Goldschleger School of Dental Medicine, Tel Aviv University, Tel-Aviv 69978, Israel; 2 Department of Oral Biology, Goldschleger School of Dental Medicine, Tel Aviv University, Tel-Aviv 69978, Israel; weinreb@post.tau.ac.il

This Special Issue entitled “Soft and Hard Tissue Regeneration” will cover both periodontal and implant therapies.

The goal of regenerative periodontal treatment is to restore functional periodontal support offering a valuable treatment alternative even for teeth with large periodontal destruction. With successful treatment, these may be successfully maintained in health for long periods. In most cases where teeth are extracted for periodontal reasons, implant therapy will demand an intermediate bone augmentation procedure. Thus, lack of sufficient bone volume may prevent placement of dental implants.

Although most bone grafts are only able to fill and maintain a space, where bone regeneration can occur (“osteoconductive”), the ideal bone graft will also promote bone production and hence osseous regeneration (“osteoinductive”).

Several bone augmentation procedures have been described, each presenting advantages and shortcomings.

The success of bone augmentation procedures depends on the presence of bone forming cells, primary wound closure over the augmented area, space creation and maintenance where bone can grow, and proper angiogenesis of the grafted area.

Factors that influence the choice of the surgical technique are the estimated duration of surgical procedure, its complexity, cost, total estimated length of procedure until the final rehabilitation may be installed, and the surgeons’ experience.

This Special Issue will have a definite clinical orientation, and will be entirely dedicated to soft and hard tissue regenerative treatment alternatives, both in periodontal and in implant therapy, discussing their rationale, indications and clinical procedures. Internationally-renowned leading researchers and clinicians will contribute with articles in their field of expertise.

## Keywords

- periodontal treatment
- dental implants
- orthodontic treatment
- complications
- pre-clinical research
- clinical research
- esthetics
- maxillary sinus
- bone augmentation
- bone grafts
- membranes
- osteoconduction
- osteoinduction
- growth factors
- therapy

